# A New Species of *Scirtothrips* Infesting *Ginkgo biloba* in Eastern China

**DOI:** 10.1673/031.012.11701

**Published:** 2012-10-10

**Authors:** Majid Mirab-balou, Xiao-li Tong, Xue-xin Chen

**Affiliations:** ^1^ Department of Entomology, College of Natural Resources and Environment, South China Agricultural University, Guangzhou 510642, China; ^2^ Institute of Insect Sciences, Zhejiang University, 866 Yuhangtang Road, Hangzhou 310058, China; ^3^ Department of Plant Protection, College of Agriculture, Ilam University, Ilam, Iran

**Keywords:** damage, ginkgo tree, Thripidae, thrips

## Abstract

A new thrips species, *Scirtothrips ginkgoe* Mirab-balou, Tong, and Chen, sp. n. (Thripidae: Thripinae) from Eastern China, collected on the leaves of *Ginkgo biloba* L. (Ginkgoaceae), is described and illustrated based on the male and female adult stage and the larva.

## Introduction

*Scirtothrips* Shull (Thripidae: Thripinae) consists of very small, active thrips that breed on the young leaves of plants, although adults may at times be found on flowers (Hoddle and Mound 2003; Hoddle et al. 2008). Several of them, such as *S. dorsalis, S. citri*, and *S. perseae*, are serious pests on perennial fruits, vegetables, and ornamental crops (Mound and Marullo 1996; Nakahara 1997; Hoddle and Mound 2003; Masumoto and Okajima 2007; Hoddle et al. 2008). This genus includes 102 described species in the world (Mound 2012), of which seven species have been recorded from China (Mirab-balou et al. 2011). Among them, only *S. dorsalis* is widely distributed in China.

**Figures 1–5.**
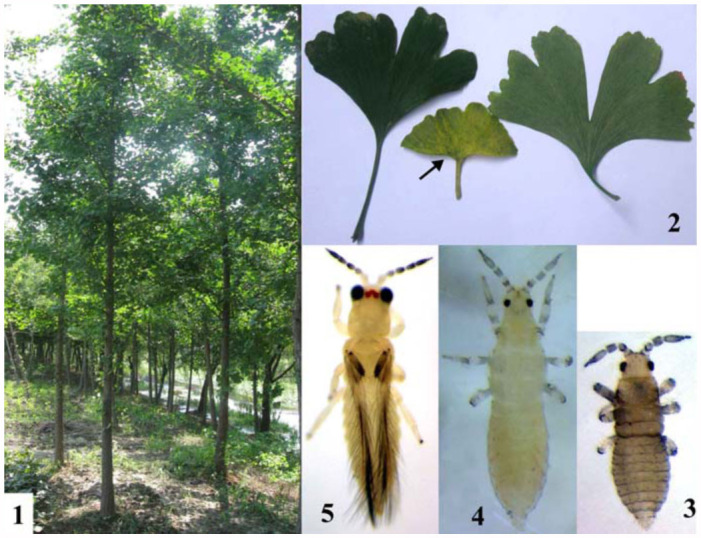
(1) Ginkgo trees; (2) Ginkgo leaves (damaged by thrips is shown in the middle); 3–5: *Scirtothrips ginkgoe* sp. n.: (3) Larva I, (4) Larva II, (5) Adult, female (80X). High quality figures are available online.

The ginkgo tree, *Gingko biloba* L. (Gingkoaceae) (Figure 1) is the oldest living tree species, with at least a 200 million year history (Shen et al. 2004). It is probably native to China, and has been widely cultivated and introduced since an early period in human history because of its various uses as food and traditional medicine (Fu et al. 1999). Extracts of Ginkgo leaves contain flavonoid glycosides and terpenoids (ginkgolides, bilobalides), and have been used pharmaceutically (Dekosky et al. 2008; Snitz et al. 2009). In this paper, we describe a previously unknown species of the genus *Scirtothrips* that was discovered damaging leaves of ginkgo trees in Hangzhou, Eastern China.

## Materials and Methods

Specimens were collected from Hangzhou, Zhejiang province, eastern China during May–August 2011. In a laboratory, thrips were prepared and mounted on glass slides using the method of Mirab-balou and Chen (2010).

Morphological terminology follows Masumoto and Okajima (2007), and Kucharczyk (2010). All descriptions, measurements, and photos were made with a Leica DM IRB microscope, a Leica MZ APO microscope with a Leica Image 1000 system. The type specimens were deposited in the Institute of Insect Sciences, Zhejiang University, Hangzhou, China. Body length is in millimeters (mm), and other measurements are in micrometers, unless otherwise stated.

***Scirtothrips ginkgoe*** Mirab-balou, Tong and Chen, sp. n. (Figures 3–11)

***Female macroptera*** (Figure 5). Distended body length 1.0–1.1 mm. Body color yellow or grayish white, with antecostal ridges of tergites pale brown and shaded on sternites; antennal segments I–II light yellow, III–VIII brown, III with pale yellow on basal half, IV–V with extreme base barely pale; forewings strongly shaded but paler toward apex.

**Figures 6–11.**
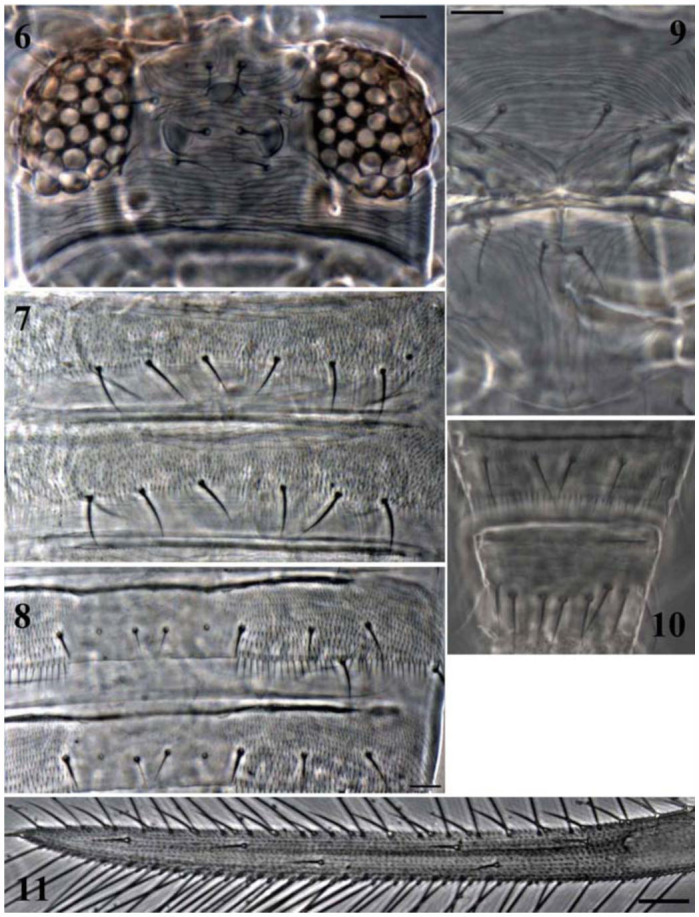
*Scirtothrips ginkgoe* sp. n.: (6) Head; (7) Abdominal sternites IV–V; (8) Abdominal tergite V; (9) Meso- and metascutum; (10) Abdominal tergites VIII–IX, male; (II) Forewing. (Scale bar = 30 microns). High quality figures are available online.

*Head* (Figure 6). Head 2.3–2.5 times as wide as long, distinctly sculptured with narrow spaced transverse striate. Two pairs of anteocellar setae present, interocellar setae situated between posterior ocelli and 1.2–1.3 times as long as distance between their bases; postocular setae pair III smaller than other setae. Compound eyes with no ommatidia strongly pigmented. Antennae 8-segmented, segment I without dorsal apical setae, III and IV each with forked sense cone; antennal segments II–VI with rows of microtrichia on both dorsal and ventral surfaces. Antennal segments I to VIII length/width ratio as follows: 0.9–1.0, 1.2–1.3, 2.4–2.5, 2.3–2.4, 2.2–2.3, 2.7–2.8, 1.1–1.2, and 2.4–2.5.

*Thorax*. Pronotum 1.9–2.1 times as wide as long, closely striate, with about 10–12 discal setae (including anteromarginals and lateralmarginals); four pairs of posteromarginal setae, B2 setae 0.4 times as long as pronotal median length and 1.6 times as long as B1. Mesonotum without campaniform sensilla anteromedially (Figure 9). Metanotal sculpture variable, transversely arcuate anteriorly, with longitudinal reticulations posteriorly; median pair of setae far behind anterior margin (Figure 9). Meso and metathoracic furcae both with spinula. Forewing first vein with three basal setae, three middle setae and three distal setae; second vein with two setae; clavus with four marginal setae and one discal setae; posteromarginal fringe cilia all straight (Figure 11).

*Abdomen*. Abdominal tergites with bases of median setae (S1) usually closer together than length of these setae (Figure 8), especially on III and IV; lateral microtrichial fields with three discal setae (Figure 8); tergites II–VII with posteromarginal microtrichia lateral to S2 setae; median part of tergites without microtrichia (Figure 8); tergite VIII with discal microtrichia present anteromedially, posteromarginal comb long and complete; tergite IX with discal microtrichia, X with no microtrichia; median setae on tergites VI–VII much longer than S1 setae on tergites II–V; abdominal tergites with antecostal ridges across full width of segment. Abdominal sternites with microtrichia extending fully across median area on posterior half (Figure 7); sternite II with two pairs of posteromarginal setae, three pairs on III–VII; median setae on sternite VII arising slightly in front of posterior margin. Ovipositor well-developed, 1.5–1.6 times as long as pronotal median length.

***Measurements*.** Distended body length 1030, width 305. Head: length 60, width 150. Compound eyes: dorsal length 55, dorsal width 42. Pronotum: median length 94, median width 176. Posteroangular setae: I 23, II 38. Forewings: length 620, width 34 at middle. Abdominal tergite IX: median length 52; S1 setae 40, S2 setae 55, S3 setae 50, middorsal setae 32. Tergite X: median length 42. Ovipositor 165 long. Antennal segments I to VIII length (width) as follows: 23 (27), 34 (27), 47 (22), 40 (22), 38 (21), 45 (18), 12 (11), and 16(8).

**Figures 12–17.**
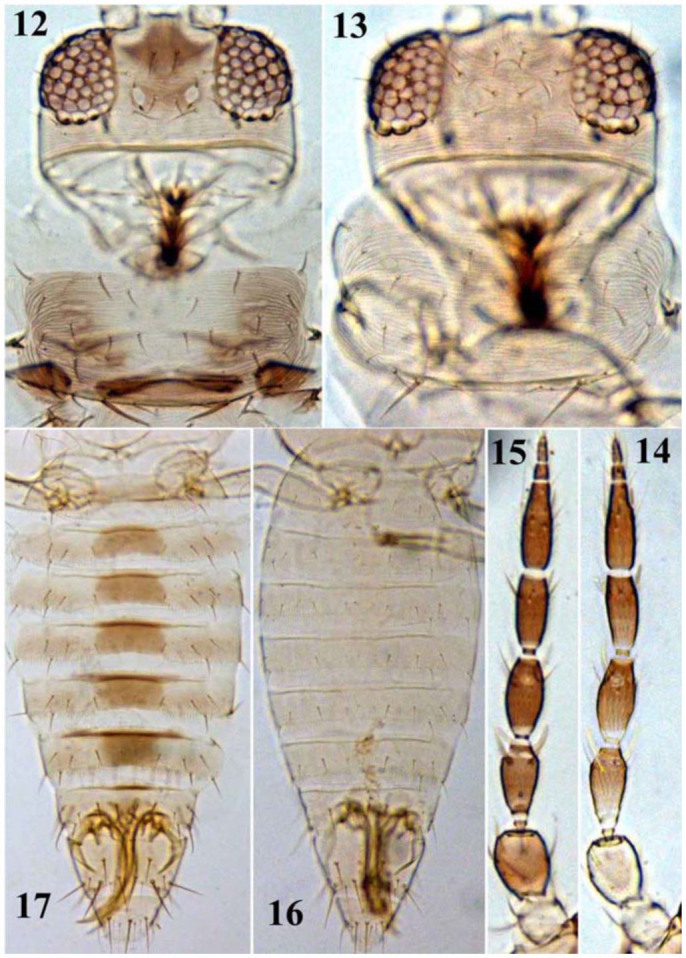
12–13: Head and pronotum: (12) *S. dorsalis*, (13) *S. ginkgoe* sp. n.; 14–15: Antenna: (14) *S. ginkgoe* sp. n., (15) *S. dorsalis*. 16–17: Abdomen: (16) *S. ginkgoe* sp. n., (17) *S. dorsalis*. High quality figures are available online.

***Male macroptera*.** Distended body length 0.8–0.9 mm. Similar to female in color and sculpture, but smaller. Abdominal tergite IX without drepanae (Figure 10); aedeagus without stout spines.

***Measurements*.** Distended body length 820–910. Head: length 65–72, width 138–143. Compound eyes: dorsal length 47–51, dorsal width 41–43. Pronotum: median length 68–72, median width 156–162. Posteroangular setae: I 13–16, setae II 23–27. Forewings: length 485–520, width 30–33.

**Figures 18–22.**
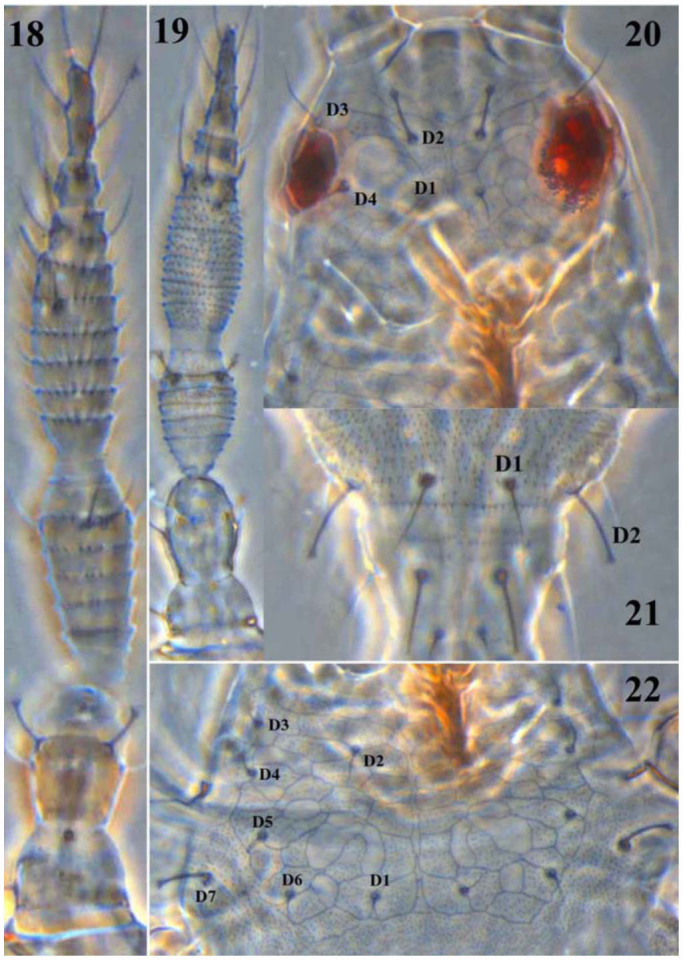
*Scirtothrips ginkgoe* sp. n.: (18) Antenna of second instar larvae; (19) Antenna of first instar larvae; 20–22: Second instar larvae: (20) Head; (21) Abdominal tergites IX–X; (22) Pronotum. (Phase contrast 3; 400X). High quality figures are available online.

***Larva*** (Figures 18–22). Second instar larva white to pale yellow; mouth-cone short; pronotum with distinctive reticulate markings; antennal segments III and IV with five and seven rings of microtrichia rows on both dorsal and ventral surfaces; body with two types of setae, i.e., acute and capitate at apex. The chaetotaxy on the body of second instar of larvae as follows: head with setae D1, D3, and D4 acute at apex while D2 capitate (D1 11 µm, D2 = 18 µm, D3 24 µm, D4 14 µm); pronotum with setae D1–D6 acute while D7 capitate (Dl 8 µm, D2 11.5 µm, D3 8 µm, D4 10.5 µm, D5 10 µm, D6 10 µm, and D7 19 µm); meso- and metanotum with acute setae; abdominal tergites II–VIII with setae D1–D3 acute at apex; tergites IX–X with setae D1 acute while D2 capitate (D1 16 µm, D2 21 µm); abdominal sternites with short acute setae.

***Material examined*.** Holotype female: **CHINA**, Zijingang Campus at Zhejiang University (30°15′ 19″ N, 120° 10′ 08″ E, 18 m. ASL), Hangzhou, Zhejiang province, from *Gingko biloba* (Gingkoaceae), 26.vi.2011, Coll. M. Mirab-Balou, (in ZJUH). Paratypes: 7 females, 3 males, 3 second instar larvae, 2 first instar larvae, collected with holotype, same data, (in ZJUH); 4 females, 1 male, **CHINA**, Tianmushan Road (near the Xixi Campus at Zhejiang University) (30° 15′ 19′ ′N, 120°10′ 08″ E, 18 m. ASL), Hangzhou, Zhejiang province, from *Gingko biloba* (Gingkoaceae), 14.vii.2011, Coll. M. Mirabbalou, (in ZJUH).

***Distribution*.** China: Zhejiang Province.

***Etymology*.** This species is named after the generic name of its recorded host, *Ginkgo*.

***Economic Importance*.** This species feeds on ginkgo foliage. The leaves become bronzed to yellow color when the thrips are found in high populations (Figure 2).

***Remarks*.** This new species is most similar to *S. dorsalis*, but is readily distinguished from the latter by the following characteristics: absence of brown markings on abdominal tergites (Figure 16) (versus the presence of brown markings medially on abdominal tergites III–VII in *S. dorsalis*) (Figure 17); antecostal ridges of tergites pale brown and shaded on sternites (versus dark brown on both tergites and sternites); head and pronotum yellow or grayish white (Figure 13) (versus head pale brown at apex of frons and pronotum with brown marking laterally in *S. dorsalis*) (Figure 12); and antennal segments I–II light yellow (Figure 14) (vs. antennal segment I pale and II–III grey in *S. dorsalis*) (Figure 15). The second instar larvae of this new species and *S. dorsalis* are similar in having reticulate sculpture on the head and pronotum, but the head with setae D4 is acute in this new species (Figure 20) while capitate in *S. dorsalis*; the body of the new species is also paler than *S. dorsalis*.

This new species is also distinguished from *S. asinus* Wang by the following characteristics: forewing second vein with two setae (versus three setae in *S. asinus*); antecostal ridge of abdominal tergites pale brown (versus dark brown in *S. asinus*); male without drepanae on abdominal tergite IX (versus with drepanae in *S. asinus*). Among remaining species recorded from China (except *S. dorsalis* and *S. asinus*), this new species is easily characterized by microtrichia extending fully across median area on posterior half of abdominal sternites, and having tergites without dark antecostal ridges. Abdominal sternites with microtrichia do not extend across median area in other remaining Chinese species.

According to the key and descriptions in Mound and Stiller (2011), this new species is similar to *S. oligochaetus* (Karny), but is readily distinguished from the latter by having three discal setae on the lateral microtrichial fields of abdominal tergites (versus 4–5 discal setae in *S. oligochaetus*); forewing brown (versus pale in *S. oligochaetus*); and sternites with microtrichia extending fully across median area on posterior half (versus with microtrichia covering median area except on anterior half in *S. oligochaetus*).
